# A case report of minimally invasive management of congenital retrocaval ureter

**DOI:** 10.1093/jscr/rjaf134

**Published:** 2025-03-12

**Authors:** Orna T Cantillon, Ibrahim Haidaran, Ned Kinnear, Derek B Hennessey

**Affiliations:** Urology Department, Mercy University Hospital, Grenville Place, Cork City T12 WE28, Ireland; Royal College of Surgeons, 123 Stephen's Green, Dublin 2 D02 YN77, Ireland; Urology Department, Austin Hospital, 145 Studley Road, Heildelberg, Melbourne, VIC 3084, Australia; Urology Department, Mercy University Hospital, Grenville Place, Cork City T12 WE28, Ireland

**Keywords:** anatomic variation, urology, congenital anomaly, minimally invasive surgery

## Abstract

Retrocaval ureter (RU) is a rare congenital malformation where the ureteric pathway is altered, passing posteriorly around the inferior vena cava (IVC). Occasionally, this leads to the IVC compressing the ureter, resulting in obstruction. In this report, we discuss a male who presented with severe right-sided flank pain and was otherwise well with no significant medical, urological, or birth history. Initial imaging revealed severe right-sided hydroureter with distinct obstruction point, and likely RU. Anecdotal and small centre cases of acute management and laparoscopic RU repair were reviewed. Initial management focused on pain control requiring a nephrostomy with subsequent transperitoneal laparoscopic dissection and repair. Operative time was 138 min with 50 mL blood loss. Post-operatively the patient recovered well and was symptom free at 6 month follow up ultrasound. Similarly, most reviewed cases chose a trans-peritoneal laparoscopic approach with good outcomes.

## Introduction

Retrocaval ureter (RU) has a prevalence of 0.13%. Its predominantly right-sided and occasionally results in clinical symptoms of hydroureteronephrosis [1]. Patients typically remain asymptomatic until the third or fourth decades of life, when obstructive symptoms may manifest [2]. This anomaly occurs due to an error in embryogenesis, and more commonly in males [3]. Anatomically, the right ureteric path is ventral to the infra-renal inferior vena cava (IVC), alterations in this result in RU. Symptoms arise when the IVC compresses the proximal ureter [4].

First reported by Hochstetter in 1893, with just over 200 cases published since [5]. Presentations vary from mild flank pain to gross haematuria or symptomatic hydronephrosis [6]. Operative repair has been documented with the predominant approach being trans-peritoneal laparoscopic ureteric dissection and re-anastomosis, newer reports of robotic laparoscopy have been described [7]. However, these reports are primarily anecdotal, with a handful of small centre studies spanning decades. We aim to report the contemporary management of a rare congenital anomaly thereby adding to guiding literature for future cases.

## Case report

A 32-year-old male referred for investigation of chronic right-side flank pain and right-sided hydronephrosis on magnetic resonance imaging of lumbar spine. With no associated haematuria. Over-the-counter analgesics were unsuccessful in curtailing the pain. The patient had no personal or family history of urolithiasis, and no significant past medical or birth history. Excessive oral fluids had caused exacerbations of pain previously.

Physical examination noted right-sided costo-vertebral angle pain. The working diagnosis was hydronephrosis secondary to a right-sided ureteric urolithiasis, as is common at this age [8]. Other potential diagnoses included pelvic-ureteric junction obstruction (PUJO), retroperitoneal fibrosis, vesicoureteric reflux, and ureteric compression due to abdominal mass/malignancy.

**Figure 1:**
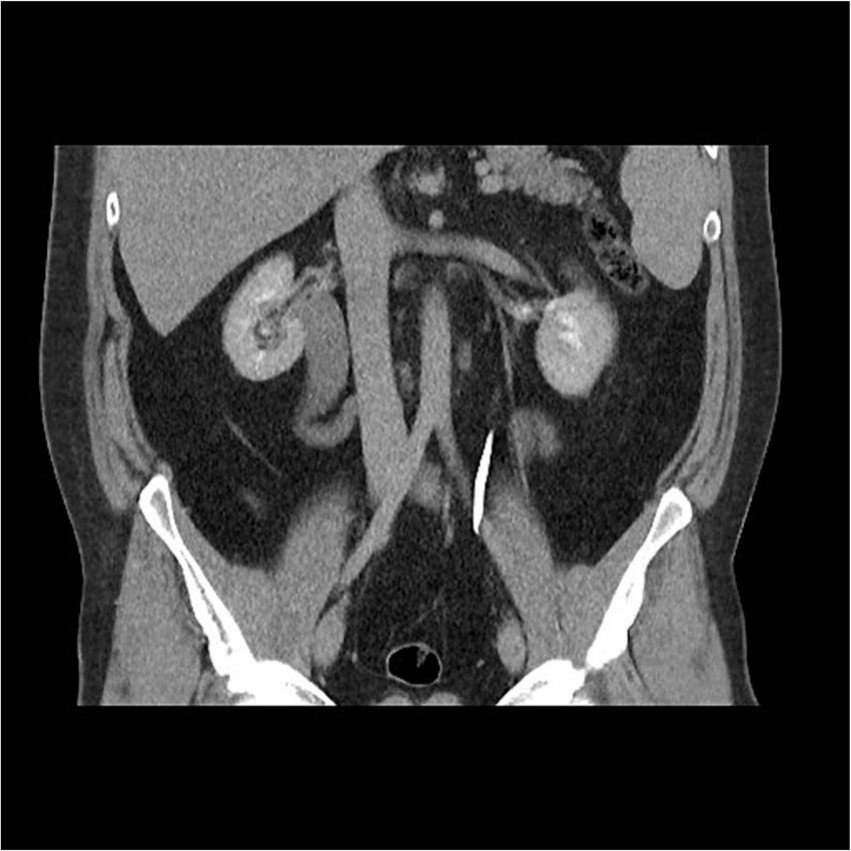
CT abdomen shows an S-shaped ureter with the right ureter passing behind the IVC.

**Figure 2:**
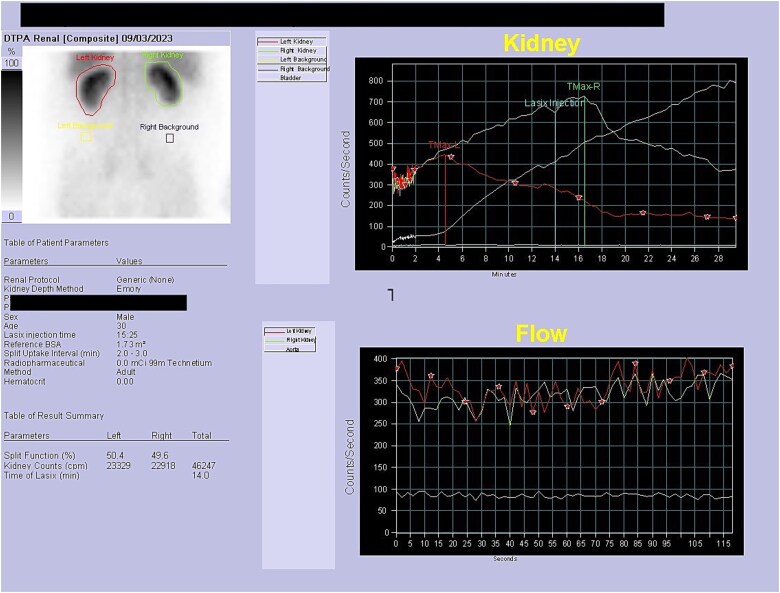
DTPA scan showing partially obstructed or hypotonic right kidney with resolution of obstruction following diuretic.

**Figure 3:**
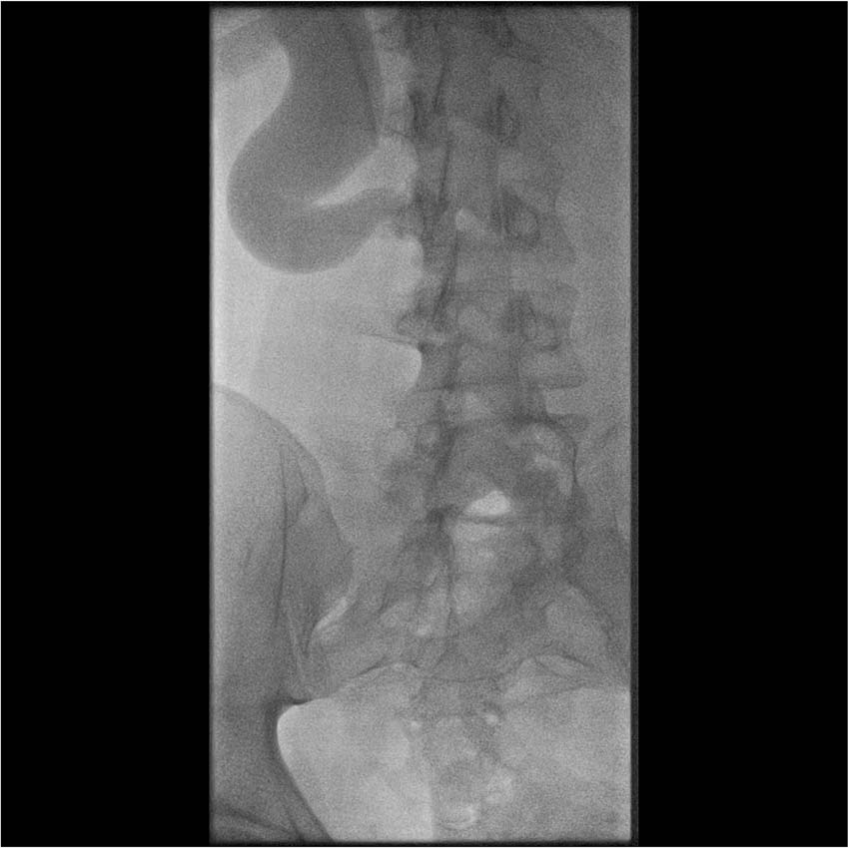
Interventional image during nephrostomy placement demonstrating S-shaped ureter and clear point of obstruction.

Initial work-up revealed microscopic haematuria and a mildly elevated creatinine of 156 μmol/L (65.4–119.3 μmol/L). A computerized tomogram (CT) urogram showed proximal hydroureteronephrosis with preserved renal cortex (Fig. 1). A diethylenetriamine pentaacetate (DTPA) renogram with diuretic showed accumulation of contrast then prompt excretion with administration of diuretic, suggesting partial obstruction or hypotonic collecting system (Fig. 2). At this point alternative diagnoses were considered, including RU. Ongoing severe right flank pain was noted despite multimodal analgesia and a right-sided nephrostomy was placed with immediate relief of pain and obstruction. Figure 3 shows the nephrostogram with S-shaped hydroureter. No further analgesia was required, and creatinine normalized (90 μmol/L). The case was discussed at a multidisciplinary meeting, wherein a unanimous diagnosis of RU was confirmed, and a plan for minimally invasive laparoscopic dissection and re-anastomosis of the ureter was made.

**Figure 4:**
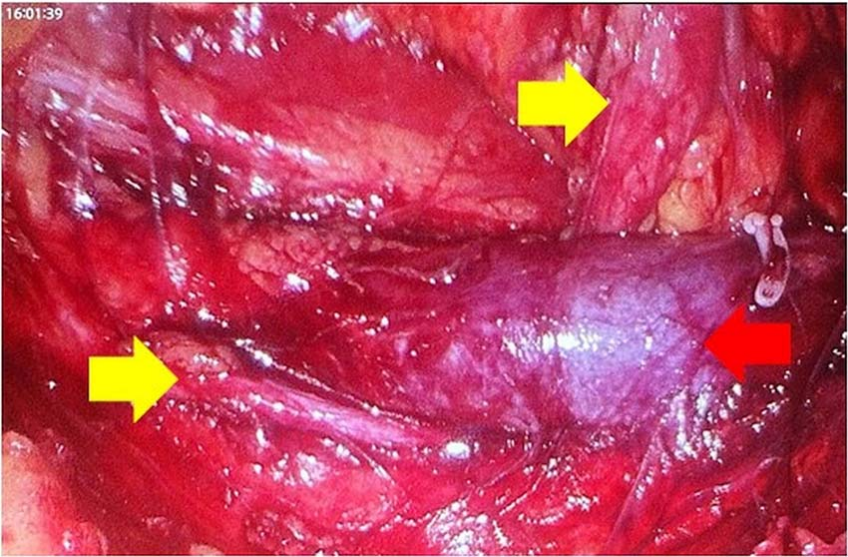
Mobilization of the ascending colon medially has exposed the IVC (red arrow) and the ureter (yellow arrows). Note how the ureter passes behind the IVC. The gonadal vein needed to be removed. Note the hem-O-Lok clip on the vein coming off the IVC.

**Figure 5:**
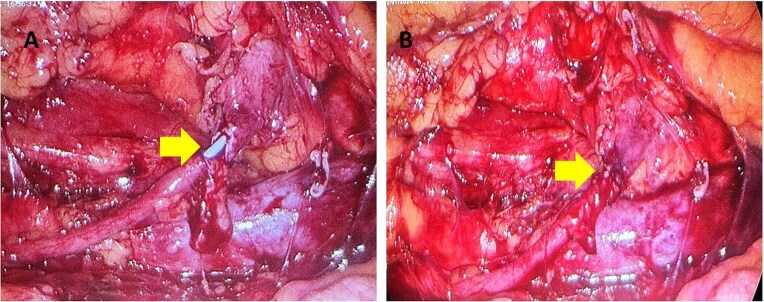
(A, B) The yellow arrow is pointing to incomplete uretero-ureteral anastomosis. The blue JJ stent is visible. In Fig. 5(B) the anastomosis is complete.

One month later a laparoscopic repair of the RU was completed. Under general anaesthetic the patient was placed right side up. A pneumoperitoneum was created with the Kii Fios first entry system and a 5 mm camera, two additional ports were used (5 mm and 12 mm). The ascending colon was mobilized medially and the IVC identified. Careful dissection allowed identification of the RU (Fig. 4). The ureter was fully mobilized behind the IVC, then divided and spatulated. Two 4.0 vicryl stay sutures were used to anastomose the apex of the spatulated ureter to proximal ureter. The posterior uretero-ureteral anastomosis was performed with interrupted 4.0 vicryl sutures. A 6 French (Fr), 24 cm JJ stent was placed into the ureter and the anterior anastomosis was performed again with interrupted 4.0 vicryl sutures (Fig. 5A and B). A 16Fr non-suction drain and a 16Fr urinary catheter were placed.

The total operative time was 138 min with a blood loss of 50 mL.

Post-operatively, the patient was allowed to eat, drink and mobilize. The catheter was removed on day two. On day three the nephrostomy was clamped with drain removal on day four. On day five the nephrostomy was removed, the patient was discharged on day six.

Over the following months, the patient recovered well with no complications. Six-week follow up appointment for JJ stent removal by flexible cystoscopy was without complication. The patient returned to his original functional baseline within three weeks post-operatively. Prospective surveillance with ultrasound at six months post-operatively revealed no adverse effects.

## Discussion

RU is a consequence of abnormal embryogenesis of the ureter and the right supra-cardinal vein. The fourth week of intrauterine development marks the beginning of the urinary tract formation, the ureters come from the mesonephros located in the nephric duct and are incorporated into the posterior bladder wall, they elongate to form anatomical ureters as the abdomen enlarges [9]. The right supra-cardinal vein, which lies dorsally, becomes the inferior vena cava in the fourth foetal month. Disruption in this structural pathway can preclude a precaval ureter. RU is classified into two categories depending on its relationship with the IVC. Type 1 has a characteristic S-shaped appearance with point of obstruction lying infero-laterally to the third lumbar vertebrae [10]. Type 2 the ureter appears sickle-cell shaped, and the obstruction lies more medially than the aforementioned [11].

Baba and colleagues reported the first case of laparoscopic treatment for RU in 1994 [12].

There are three main minimally invasive approaches to RU repair described in the literature; retroperitoneal, trans-peritoneal, and robotic. The former approach described by Liu *et al*., concluded that retreoperitoneoscopic ureteroplasty be recommended as first line with a mean operative time of 103 min [13]. Trans-peritoneal appears to be the most utilized in the literature to date with reports of successful laparoscopic ureteroplasty or pyeloplasty, including via robotic approach [7, 10, 14, 15]. Dogan *et al.* and Gupta *et al.* took a transperitoneal approach reporting operative times of 96.6 min, and 210 min, respectively, with 71 mL of blood loss in the former [10, 14].

Another nine patient study followed a trans-peritoneal approach and reported a mean operative time of 135 min with ˂60 mL of blood loss in the former [15]. Both approaches show relatively similar operative times and blood loss. The retroperitoneal method used was a three-port approach with visualization, dissection, and restoration of anatomical position with JJ stent insertion. Importantly, all studies reported symptom-free patients at three or six month follow-up appointments [7, 10, 13–15]. Few reports of robotic approach have been described in the literature with a recent case by Maestroni *et al.* reporting a similar operative time of 140 min with good surgical outcomes [7]. Importantly, these reports focus on the surgical repair and rarely discuss the initial acute management therefore there is little to suggest preferred use of nephrostomy over stenting for initial relief of pain and obstruction.

Minimally invasive surgical repair of RU is highly successful with few post-operative complications. Our study corroborates these findings and adds to the supporting literature for the trans-peritoneal approach with dissection, and restoration of anatomy. Of note, there are no accepted guidelines for RU repair outside of these cases emphasizing the importance of scientific reporting of case-specific management for safe and effective management of rare anomalies.
